# Projected Prevalence of Actionable Pharmacogenetic Variants and Level A Drugs Prescribed Among US Veterans Health Administration Pharmacy Users

**DOI:** 10.1001/jamanetworkopen.2019.5345

**Published:** 2019-06-07

**Authors:** Catherine Chanfreau-Coffinier, Leland E. Hull, Julie A. Lynch, Scott L. DuVall, Scott M. Damrauer, Francesca E. Cunningham, Benjamin F. Voight, Michael E. Matheny, David W. Oslin, Michael S. Icardi, Sony Tuteja

**Affiliations:** 1 US Department of Veterans Affairs, VA Informatics and Computing Infrastructure, Salt Lake City Health Care System, Salt Lake City, Utah; 2 Center for Healthcare Organization and Implementation Research, US Department of Veterans Affairs, Boston Healthcare System, Boston, Massachusetts; 3 US Department of Veterans Affairs, Edith Nourse Rogers Memorial Veterans Hospital, Bedford, Massachusetts; 4 College of Nursing and Health Sciences, University of Massachusetts, Boston; 5 Department of Internal Medicine, University of Utah School of Medicine, Salt Lake City; 6 Perelman School of Medicine, University of Pennsylvania, Philadelphia; 7 Corporal Michael Crescenz Department of Veterans Affairs Medical Center, Philadelphia, Pennsylvania; 8 US Department of Veterans Affairs Center for Medication Safety, Pharmacy Benefits Management Services, Hines, Illinois; 9 Geriatrics Research Education and Clinical Care Center, US Department of Veterans Affairs Tennessee Valley Healthcare System, Nashville; 10 Department of Biomedical Informatics, Vanderbilt University Medical Center, Nashville, Tennessee; 11 US Department of Veterans Affairs Iowa City Healthcare System, Iowa City, Iowa; 12 US Department of Veterans Affairs National Office of Pathology and Laboratory Medicine, Iowa City, Iowa; 13 Carver College of Medicine, University of Iowa, Iowa City

## Abstract

**Importance:**

Implementation of pharmacogenetic testing to guide drug prescribing has potential to improve drug response and prevent adverse events. Robust data exist for more than 30 gene-drug pairs linking genotype to drug response phenotypes; however, it is unclear which pharmacogenetic tests, if implemented, would provide the greatest utility for a given patient population.

**Objectives:**

To project the proportion of veterans in the US Veterans Health Administration (VHA) with actionable pharmacogenetic variants and evaluate how testing might be associated with prescribing decisions.

**Design, Setting, and Participants:**

This cross-sectional study included veterans who used national VHA pharmacy services from October 1, 2011, to September 30, 2017. Data analyses began April 26, 2018, and were completed February 6, 2019.

**Exposures:**

Receipt of level A drugs based on VHA pharmacy dispensing records.

**Main Outcomes and Measures:**

Projected prevalence of actionable pharmacogenetic variants among VHA pharmacy users based on variant frequencies from the 1000 Genomes Project and veteran demographic characteristics; incident number of level A prescriptions, and proportion of new level A drug recipients projected to carry an actionable pharmacogenetic variant.

**Results:**

During the study, 7 769 359 veterans (mean [SD] age, 58.1 [17.8] years; 7 021 504 [90.4%] men) used VHA pharmacy services. It was projected that 99% of VHA pharmacy users would carry at least 1 actionable pharmacogenetic variant. Among VHA pharmacy users, 4 259 153 (54.8%) received at least 1 level A drug with 1 188 124 (15.3%) receiving 2 drugs, and 912 189 (11.7%) receiving 3 or more drugs. The most common incident prescriptions during the study were tramadol (923 671 new recipients), simvastatin (533 928 new recipients), citalopram (266 952 new recipients), and warfarin (205 177 new recipients). Gene-drug interactions projected to have substantial clinical impacts in the VHA population include the interaction of *SLCO1B1* with simvastatin (1 988 956 veterans [25.6%]), *CYP2D6* with tramadol (318 544 veterans [4.1%]), and C*YP2C9* or *VKORC1* with warfarin (7 163 349 veterans [92.2%]).

**Conclusions and Relevance:**

Clinically important pharmacogenetic variants are highly prevalent in the VHA population. Almost all veterans would carry an actionable variant, and more than half of the population had been exposed to a drug affected by these variants. These results suggest that pharmacogenetic testing has the potential to affect pharmacotherapy decisions for commonly prescribed outpatient medications for many veterans.

## Key Points

**Question:**

What is the potential impact of implementing pharmacogenetic testing for gene-drug interactions with a high level of evidence (level A)?

**Findings:**

This cross-sectional study of more than 7.7 million US veterans used variant frequencies from the 1000 Genomes Project and veteran demographic characteristics to estimate that 99% of veterans who use the Veteran Health Administration carry at least 1 actionable pharmacogenic variant. Analysis of Veterans Health Administration pharmacy records during a 6-year period suggested that 2.9 million veterans (37%) started treatment with at least 1 level A drug, with 25% of them receiving 2 level A drugs and 12% receiving 3 or more level A drugs.

**Meaning:**

Pharmacogenetic testing has the potential to inform pharmacotherapy decisions for most veterans.

## Teaser

This cross-sectional study uses 1000 Genomes Project data and US Veterans Health Administration demographic characteristics to project the number of veterans who carry an actionable pharmacogenetic variant and who have been prescribed 1 or more level A drugs that have well-established evidence of pharmacogenetic interactions.

## Introduction

Pharmacogenetic variants contribute to individual differences observed in the response to medications and risk of adverse drug reactions.^1,2^ Approximately 10% of the 1200 medications approved by the US Food and Drug Administration contain pharmacogenetic information in the drug label.^3^ Clinical knowledge of pharmacogenetic variants has the potential to affect a patient’s care throughout life since they are inherited and therefore do not change. Recent large-scale genotyping and sequencing studies have found that more than 95% of patients carry at least 1 pharmacogenetic variant that would influence medication-related outcomes and would be deemed actionable.^4,5,6^ Overall, pharmacogenetics has potential to provide a safer and more effective process for prescribing medication, ultimately improving health outcomes and reducing health care costs.^7^ Yet translation of this knowledge into clinical care has been slow.^8^

Two collaborative initiatives are leading the efforts in curating and disseminating information about pharmacogenetics: the National Institutes of Health–funded Pharmacogenomic Knowledgebase^1^ and the Clinical Pharmacogenetics Implementation Consortium (CPIC).^9^ To accelerate the translation of test results into actionable prescribing decisions, CPIC publishes peer-reviewed, evidenced-based guidelines for specific medications. There are more than 30 gene-drug interactions classified by CPIC as *level A*, indicating a high level of evidence from well-designed, well-conducted studies, including the commonly prescribed drugs simvastatin,^10^ clopidogrel,^11^ and codeine.^12^ One gap in pharmacogenetic understanding is that CPIC guidelines provide no guidance regarding when to order pharmacogenetic tests.

A barrier to pharmacogenetic implementation in practice is uncertainty about which pharmacogenetic tests have the greatest clinical utility for a patient population. A thorough assessment of the prevalence of pharmacogenetic variants and prescribing patterns for medications affected by pharmacogenetic variants in the target population may help to identify tests with greater utility and guide implementation efforts to increase adoption in clinical settings. The Veterans Health Administration (VHA) is the largest integrated health care system in the United States, including 9.1 million veterans enrolled in 2016 and a nationally integrated electronic health record (EHR),^13^ with the potential to link laboratory test results to medication prescription, dispensation, and administration. The purpose of this study is to evaluate the potential impact of adopting pharmacogenetic tests for level A drugs within the VHA. We estimated the prevalence of actionable pharmacogenetic variants among veterans, quantified the number of unique veterans who were prescribed level A medications, and projected the potential clinical impact that knowledge of actionable pharmacogenetic variants could have on prescribing decisions for veterans.

## Methods

This study was approved by the US Department of Veterans Affairs (VA), Bedford, Massachusetts, and University of Utah institutional review boards and research and development committee and received Health Insurance Portability and Accountability Act authorization. A waiver of consent was granted because the research involved no more than minimal risk to the participants, was performed using data collected during routine clinical care, and could not practically be carried out without the waiver of consent. The study followed the Strengthening the Reporting of Observational Studies in Epidemiology (STROBE) reporting guideline for cross-sectional studies.

### Demographic Characteristics

Demographic characteristics of veterans who received care in the VHA from October 1, 2011, to September 30, 2017, were extracted from Observational Medical Outcomes Partnership tables from the VA Corporate Data Warehouse within the VA Informatics and Computing Infrastructure.^14^ Unique VHA users were identified based on any record for VHA inpatient and outpatient care, laboratory tests, and prescriptions (eTable 1 in the Supplement). We identified VHA pharmacy users with at least 1 medication record during the study using the Observational Medical Outcomes Partnership Drug Exposure table, a curated, national data set for all medications dispensed through VHA pharmacies in outpatient and inpatient settings. Patients were included in analysis based on the existence of at least 1 record for a level A drug during the study. Patients were qualified as new drug recipients only for the year of the first level A drug prescription in the period. For patients with a first prescription in 2012, we examined their prescription records from the prior year; patients with a prescription for that drug in 2011 were then excluded from the new level A drug recipients in 2012.

### Statistical Analysis

Data analyses began April 26, 2018, and were completed February 6, 2019. Analyses were conducted based on the following assumptions: (1) genotype-guided medication prescribing is not widely performed within the VHA; (2) pharmacogenetic variants are in Hardy-Weinberg equilibrium within the VHA population; and (3) pharmacogenetic carrier status does not affect the initial need for the medication.^15^ We collected the allele frequencies of pharmacogenetic variants with a level A gene-drug interaction for ancestry-specific populations from the 1000 Genomes Project.^16,17,18^ Variants in the same gene were treated as mutually exclusive (eTable 2 in the Supplement), and the frequency of the wild-type allele was calculated as 1 minus the sum of the actionable variant frequencies within that population. Numbers of actionable genotypes (ie, number of homozygous, homozygous and heterozygous, or carriers of the variants, as applicable) were calculated for each population (eTable 3 in the Supplement).

For the gene *G6PD* on chromosome X, the frequency of actionable genotypes was estimated separately by sex (eTable 3 in the Supplement). For the genes with high polymorphism, ie, *CYP2C9*, *CYP2C19*, and *CYP2D6,* we collected the distribution of functional phenotypes classified as actionable by CPIC (eTable 4 in the Supplement), which included variations in *CYP2D6* copy number.^12,19,20^

To approximate the diversity of the VHA population (15% African ancestry), we weighted the known ancestry-specific variant frequency from 1000 Genomes Project with the racial diversity in the VHA population (eTable 1 in the Supplement). Alternative population models were tested in sensitivity analyses and yielded similar estimates for the pharmacogenetic variant prevalence (eMethods, eTable 5, eTable 6, and eFigure in the Supplement). Finally, the proportion of veterans who would carry at least 1 actionable variant was estimated as 1 minus the probability of having a wild-type genotype for all genes analyzed (eMethods and eTable 7 in the Supplement).

We calculated the overall proportion of veterans prescribed a level A drug among unique VHA pharmacy users and among new recipients of level A drugs. For the top 10 level A drugs newly prescribed to unique recipients, we estimated the proportion of drug recipients with actionable pharmacogenetic variants as the product of the reported frequency of actionable phenotypes by the number of new recipients of that drug. We also reported the projected number of patients receiving clopidogrel within 30 days after a percutaneous coronary intervention for whom clinical recommendations are strongest (eMethods and eTable 8 in the Supplement).

We estimated the absolute number of patients newly exposed to medications and at risk of drug nonefficacy or toxic effects based on the frequencies of projected phenotypes. We limited our analyses to the medications with a strong CPIC level A phenotype-based recommendation that the patient be prescribed alternative or dose-adjusted therapy (eTable 9 and eTable 10 in the Supplement).

## Results

There were 7 769 359 veterans who used VHA pharmacy services from October 1, 2011, to September 30, 2017. The mean (SD) age at the start of the study was 58.1 (17.8) years; 7 021 504 veterans (90.4%) were men. The cohort included 5 153 274 white veterans (66.3%), 1 195 906 African American veterans (15.4%), and 450 692 Hispanic veterans (5.8%) (eTable 1 in the Supplement). Table 1 shows the expected prevalence of actionable genotypes among VHA pharmacy users for each gene. Individual allele frequencies for each race/ethnic group and calculations for actionable variants are provided in eTable 2 and eTable 3 in the Supplement. The most prevalent variant was in the *IFNL3* (*IL28B*) gene (rs12979860; Table 2), which influences patient response to anti–*hepatitis C virus* medication peginterferon.^21^ After tabulating the frequencies of actionable variants, we estimated that 99% of the veterans receiving care in VHA would have at least 1 pharmacogenetic variant (eTable 7 in the Supplement). Our estimates based on population-specific allele frequencies are consistent with previous studies directly genotyping^4^ or sequencing large biobank populations,^5^ demonstrating that pharmacogenetic variants are found in more than 90% of the population.

**Table 1.** Projected Frequency of Actionable Pharmacogenetic Variants Among Veterans Health Administration Pharmacy Users

Gene	Allele	Effect	Population With Actionable Genotypes, No. (%)^a^	Drugs Affected
*CYP2C9*	*2,*3,*5,*6,*8,*11	Decreased function	2 633 813 (33.9)	Warfarin, phenytoin
*VKORC1*^19^	1639G>A	Increased warfarin sensitivity	4 529 536 (58.3)	Warfarin
*CYP2C19*	*2, *3, *4, *8	Decreased function	2 035 572 (26.2)	Clopidogrel, citalopram, escitalopram, amitriptyline
	*17	Increased function	3 348 594 (43.1)	Voriconazole
*CYP2D6*^12^	*3, *4, *5, *6, *9, *10, *17, *29, *41	Decreased or no function	318 544 (4.1)	Codeine, tramadol, fluvoxamine, paroxetine, nortriptyline, ondansetron
	Gene duplication	Increased function	264 158 (3.4)	Tamoxifen
*CYP3A5*^43^	*1	Dosage increase recommended	1 926 801 (24.8)	Tacrolimus
*SLCO1B1*	*5	Increased myopathy risk	1 988 956 (25.6)	Simvastatin
*UGT1A1*	*80	Decreased function	870 168 (11.2)	Atazanavir, irinotecan
*TPMT*	*2, *3	No function	450 623 (5.8)	Azathioprine, mercaptopurine, thioguanine
*DPYD*	*2A, D949V	No function or reduced function	69 924 (0.9)	Capecitabine, fluorouracil
*G6PD*^44^	202A/376G	Deficient	380 699 (4.9)	Rasburicase
*IFNL3*^21^	rs12979860	Unfavorable response	6 433 029 (82.8)	Pegylated interferon
HLA-A^45^	*31:01	Hypersensitivity reaction	372 929 (4.8)	Carbamazepine, oxcarbazepin
HLA-B^45^	*57:01	Hypersensitivity reaction	435 084 (5.6)	Abacavir, phenytoin
	*58:01	Severe cutaneous adverse reactions	295 236 (3.8)	Allopurinol, carbamazepine, oxcarbazepine
	*15:02	Stevens-Johnson syndrome or toxic epidermal necrolysis	5515 (0.1)	

^a^ Based on 7.8 million veterans using Veterans Health Administration pharmacy services from October 1, 2011, to September 30, 2017, and estimating the population diversity as 15% African ancestry and 85% European ancestry. Frequencies of actionable genotypes were calculated using the frequency of variants in each ancestry group, except for *CYP2D6* gene duplication, in which frequency of actionable phenotypes was used.

**Table 2.** Veterans Health Administration Pharmacy Users Prescribed Level A Drugs

Drug	Drug Class	No. (%) (N = 7 769 359)	
		All Drug Recipients	New Drug Recipients
≥1 Level A drug	Any	4 259 153 (54.8)	2 943 872 (37.9)
Simvastatin	Statin	1 925 052 (24.8)	533 928 (6.9)
Tramadol	Opioid	1 308 595 (16.8)	923 671 (11.9)
Ondansetron	Oncology	702 244 (9.0)	604 226 (7.8)
Codeine	Opioid	680 527 (8.8)	528 159 (6.8)
Citalopram	Antidepressant	569 668 (7.3)	266 952 (3.4)
Clopidogrel	Antiplatelet	560 001 (7.2)	338 295 (4.4)
Allopurinol	Gout	408 862 (5.3)	215 055 (2.8)
Warfarin	Anticoagulant	385 821 (5.0)	205 177 (2.6)
Amitriptyline	Antidepressant	257 092 (3.3)	174 693 (2.2)
Paroxetine	Antidepressant	214 166 (2.8)	138 183 (1.8)
Fluorouracil	Oncology	192 482 (2.5)	160 356 (2.1)
Escitalopram	Antidepressant	176 907 (2.3)	170 690 (2.2)
Nortriptyline	Antidepressant	123 001 (1.6)	88 551 (1.1)
Carbamazepine	Anticonvulsant	58 304 (0.8)	32 868 (0.4)
Phenytoin	Anticonvulsant	43 348 (0.6)	15 556 (0.2)
Tacrolimus	Immunosuppressant	39 168 (0.5)	26 487 (0.3)
Ribavirin	Antiviral	35 744 (0.5)	31 606 (0.4)
Oxcarbazepine	Anticonvulsant	18 763 (0.2)	15 213 (0.2)
Azathioprine	Immunosuppressant	15 769 (0.2)	9283 (0.1)
Capecitabine	Oncology	11 994 (0.2)	9677 (0.1)
Peginterferon alfa-2a	Antiviral	10 348 (0.1)	6437 (0.08)
Abacavir	Antiviral	9716 (0.1)	6263 (0.08)
Atazanavir	Antiviral	6495 (0.08)	1709 (0.02)
Voriconazole	Antifungal	4603 (0.06)	3785 (0.05)
Tamoxifen	Oncology	4374 (0.06)	3159 (0.04)
Fluvoxamine	Antidepressant	3842 (0.05)	2460 (0.03)
Mercaptopurine	Immunosuppressant	3566 (0.05)	1996 (0.03)
Rasburicase	Oncology	1637 (0.02)	1444 (0.02)
Peginterferon alfa-2b	Antiviral	NR^a^	NR^a^
Irinotecan	Oncology	NR^a^	NR^a^
Thioguanine	Immunosuppressant	NR^a^	NR^a^

Abbreviation: NR, not reported.

^a^ Number less than 1000.

**Figure 1.**
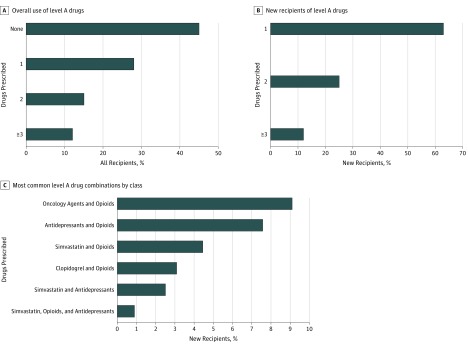
Use of Level A Drugs and Combinations Among Veterans Health Administration Pharmacy Users from October 1, 2011, to September 30, 2017 A, Proportion of Veterans Health Administration pharmacy users prescribed 1 or more level A drugs. B, Proportion of Veterans Health Administration pharmacy users newly prescribed 1 or more level A drugs. C, Proportion of new drug recipients receiving the most common combinations of level A drugs by drug classes.

Pharmacogenetic variants are considered actionable only if carriers are exposed to the associated medications. In our study, 7 769 359 veterans who received care in the VHA had at least 1 medication record, and of those VHA pharmacy users, 4 259 153 (54.8%) had at least 1 record for a level A drug (Table 2). The most common level A drug prescribed in the VHA system was simvastatin, prescribed to 1 925 052 veterans (24.8%), and exposure to multiple level A drugs was frequent (Figure 1A). During the study, 2 943 872 veterans (37.9%) received a new prescription for a level A drug, with the most frequent being tramadol, prescribed to 923 671 veterans (11.9%). Additionally, a substantial proportion of new level A drug recipients received new prescriptions for multiple level A drugs in the same year: 726 502 (24.7%) were newly prescribed 2 level A drugs and 356 685 (12.1%) were prescribed 3 or more level A drugs (Figure 1B). The most frequent combinations of drugs included opioids, oncology agents, simvastatin, antidepressants, and clopidogrel (Figure 1C).

**Figure 2.**
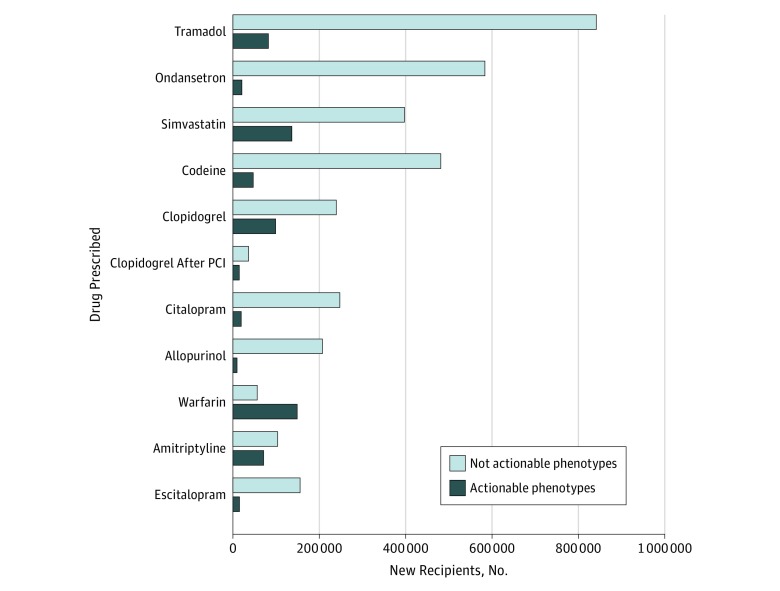
Projected Numbers of New Drug Recipients With Actionable and Nonactionable Phenotypes for the Top 10 Level A Drugs Projections based on the numbers of Veterans Health Administration pharmacy users receiving a new prescription for each drug from October 1, 2011, to September 30, 2017. Numbers are presented for all patients receiving clopidogrel and for patients receiving clopidogrel after a percutaneous coronary intervention (PCI) because of the larger clinical impact of the pharmacogenetic variant for this indication.

We estimated the proportion of individuals potentially at risk for adverse outcomes secondary to a gene-drug interaction by determining the prevalence of actionable genotypes in patients exposed to 1 of the top 10 level A drugs (Figure 2) (eTable 8 in the Supplement). For example, among 923 671 veterans newly receiving tramadol for analgesia, 82 092 (8.9%) are projected to have an inadequate response to therapy. Among 533 928 patients newly prescribed simvastatin, 136 599 (25.6%) are estimated to carry the rs4149056 variant in *SLCO1B1,* which would place them at risk of simvastatin-induced myopathy, and 32 010 simvastatin recipients with projected actionable genotypes were prescribed an 80-mg initial dose, putting them at higher risk (eTable 8 in the Supplement).

**Figure 3.**
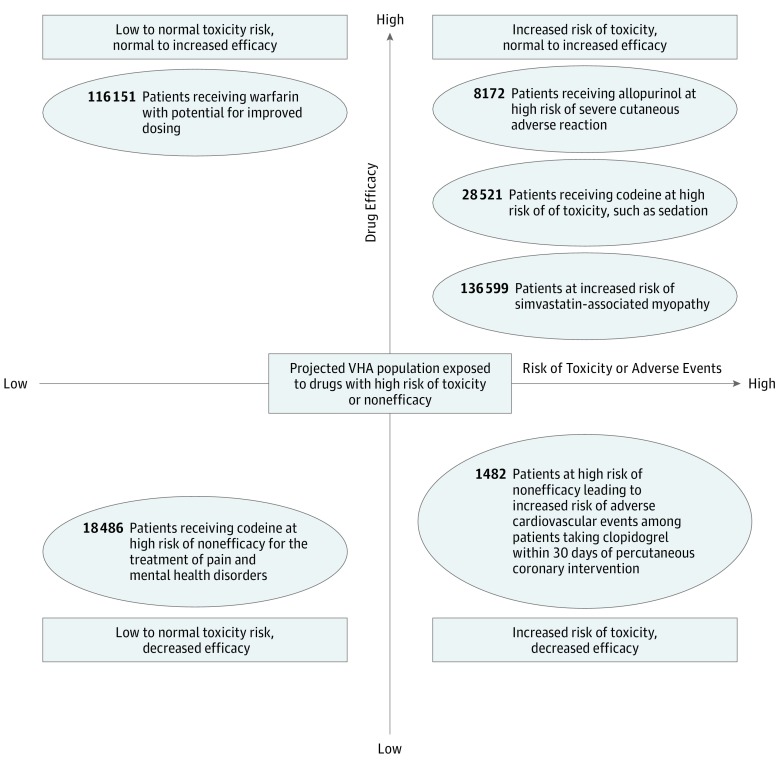
Projected Veterans Health Administration (VHA) Population Exposed to a Drug With High Risk of Toxic Effects or Nonefficacy from October 1, 2011, to September 30, 2017 Medications with a strong level A recommendation to either avoid or adjust the dose based on available pharmacogenetic test results are included. The x-axis depicts the increasing risk of toxic effects or adverse drug reaction in response to drug exposure for patients with select phenotypes. The y-axis depicts the spectrum of anticipated efficacy of the drug for patients with select phenotypes—those with certain phenotypes are at higher risk of drug nonresponse. The number of patients projected to be carriers of the genetic variant or specific phenotype is based on the numbers of new drug recipients from October 1, 2011, to September 30, 2017.

To assess the clinical effect of a particular genotype on drug response, both the translation from genotype to pharmacogenetic phenotypes and the pharmacological characteristics of the drug are important to consider. Figure 3 describes the number of veterans exposed to medications on our list of top 10 level A drugs and at high risk of therapeutic failure or toxic effects. For prodrugs like codeine, cytochrome P450 2D6, encoded by *CYP2D6*, converts codeine to morphine, a metabolite with greater activity than the parent drug. Among 528 159 veterans prescribed codeine-containing medications, 18 486 (3.5%) were predicted to be *CYP2D6* ultrarapid metabolizers and at high risk of toxic effects, and 28 521 (5.4%) were estimated to be *CYP2D6* poor metabolizers and may not receive therapeutic benefit. Among 215 055 veterans who were prescribed allopurinol, 8172 (3.8%) are estimated to carry the HLA-B*58:01 allele, which increases the risk of severe cutaneous adverse reactions, including Stevens-Johnson syndrome and toxic epidermal necrolysis. Additionally, an estimated 116 151 veterans (66.6%) of European ancestry were projected to carry genetic variants that could help to guide warfarin dosing, increasing the potential for drug efficacy and decreasing the risk of drug toxic effects (eTable 10 in the Supplement); veterans of African ancestry also are likely to have variants that could guide dosing, although different variants are likely to influence warfarin response in this population.^22,23^ Therefore, pharmacogenetic tests can be used clinically to predict a broad range of therapeutic effects.

## Discussion

To our knowledge, this is the first study to provide a reliable estimate of the number of veterans who could benefit from implementation of pharmacogenetic testing in clinical care. Almost all veterans carry at least 1 genetic variant that could influence pharmacotherapy decisions if they are prescribed a level A drug. Additionally, more than one-third of the 7.8 million veterans who received medications from the VHA pharmacy from October 1, 2011, to September 30, 2017, were newly prescribed at least 1 level A drug. These medications encompass drug classes commonly prescribed in primary care settings, such as statins, opioid analgesics, and antidepressants. Among those new level A drug recipients, 38% were prescribed more than 1 level A drug, suggesting that an initial testing for a panel of relevant pharmacogenetic variants may inform pharmacotherapy decisions over the entire course of care in the VHA. This study provides information that can help VHA leadership develop a strategy and framework for comprehensive clinical implementation of CPIC guidelines.

Our findings are largely consistent with 2 previous studies that quantified the association of pharmacogenetic information with outcomes in large health care systems.^24,25^ A 2012 study by Schildcrout et al^24^ showed that among 52 942 participants who received at least 1 of 56 medications with pharmacogenetic associations listed on the drug labels by the Food and Drug Administration, 65% were prescribed at least 1 drug during a 5-year period, and 12% were prescribed 4 or more. Based on the event probability of 6 selected severe adverse events, approximatively 400 events were potentially preventable by preemptive pharmacogenetic testing, and the medications associated with the greatest risk included clopidogrel, abacavir, azathioprine, simvastatin, tamoxifen, and warfarin. Drug exposure in the study by Schildcrout et al^24^ was higher than in our study because they used a larger list of medications, while we focused on the 30 drugs with the highest level of evidence (CPIC level A). Because of this difference, our estimates may underestimate the prescribing of drugs with lower levels of evidence or newly emerging evidence associated with pharmacogenetic variants. A 2016 study by Samwald et al^25^ that examined approximately 73 million medical records from patients enrolled in private insurance, Medicare Supplemental, or Medicaid during a 4-year period found that half of the population had received at least 1 drug affected by pharmacogenetic variants, while one-third had received 2 or more drugs. These estimates are consistent with our study in the VHA population. These results suggest that exposure to multiple drugs affected by pharmacogenetic variants is common in the population and that testing for a panel of pharmacogenetic variants may help to prevent serious adverse events.

There are 2 main models that could be used to test patients for pharmacogenetic variants. First, patients could be tested reactively, when the clinician considers ordering a drug with actionable pharmacogenetics. While this system allows clinicians to pick pharmacogenetic tests with higher levels of evidence tailored to the patient, it also has several limitations. Clinicians must be ready and able to order the tests, and the test results must be returned in a timely manner to avoid delays in prescribing.^26^

An alternative to this model is preemptive testing. Several centers have launched preemptive pharmacogenetic testing programs that target patients likely to receive drugs impacted by pharmacogenetic variants.^27,28,29,30,31^ The benefit of this approach is that genotyping is performed before the drug is needed, and results are stored in the EHR and available at the time of prescribing. Vanderbilt University has incorporated preemptive pharmacogenetic results into their EHR system for 5 gene-drug pairs: *CYP2C19* with clopidogrel, *CYP2C9 *or* VKORC1* with warfarin, *SLCO1B1* with simvastatin*, TPMT* with thiopurines, and *CYP3A5* with tacrolimus.^4^ Clinically actionable variants trigger a clinical decision support system in the EHR to guide drug selection and dosing. Similarly, the Mayo Clinic offers preemptive pharmacogenetic testing (*CYP2D6* genotyping and targeted sequencing of 84 pharmacogenetic genes) to biobank participants at high likelihood to initiate a statin treatment within 3 years.^29^ In that system, real-time clinical decision support integrated into the EHR flags specific gene-drug interactions and provides pharmacotherapy recommendations. This system takes into the account the complexity of information, as different gene variants may influence the same drugs (eg, *CYP2C9 *or *VKORC1 *with warfarin*)* or a single variant can influence response to several drugs (eg, *CYP2C19* with clopidogrel or escitalopram). One challenge is the ability for clinical decision support tools to stay current with the emerging knowledge base as more evidence and guidelines become available and prescribing patterns change.^32^

The VHA has been on the forefront of adopting precision medicine innovations in the clinical care of veterans. For example, next-generation sequencing is offered to veterans diagnosed with cancer,^33^ and pharmacogenetic testing of the HLA-B*57:01 allele prior to prescribing abacavir is already standard of care. Our study suggests that further expansion of pharmacogenetic testing may benefit many veterans and should be considered for the next genomic medicine implementation in the VHA. As the VHA is in the process of deploying a new EHR, the timing of clinical pharmacogenetic implementation may be ideal. Establishing a robust informatics pipeline linking pharmacogenetic test results from the laboratory to the pharmacy to flag gene-drug interactions would facilitate appropriate selection and dose of medications dispensed by pharmacists. Full integration of pharmacogenetic laboratory results and pharmacy data could greatly reduce severe adverse events caused by gene-drug interactions. A partnership of laboratory medicine and pharmacy already exists for the treatment of hepatitis C to guide the use of new direct-acting antiviral drugs informed by viral genetics. In 2014, VHA laboratories began performing resistance testing and, in partnership with the pharmacy department, more than 50 000 veterans have been successfully tested and treated for hepatitis C within a 2-year period.^34^ From a clinical laboratory technology perspective, it would not be not difficult to expand capabilities from viral genetics to pharmacogenetics.

The VHA is also an international leader in genomic medicine research with the Million Veteran Program.^35^ More than 700 000 veterans have undergone genome-wide evaluation on a custom genotyping array,^35^ which contains several of the same clinically actionable pharmacogenetic variants described in our study. Pharmacogenetic information from this representative cohort, combined with EHR and pharmacy data, is expected to fill a gap in evidence on pharmacogenetic impact in diverse populations. However, for these data to be leveraged for clinical care, they would need to be validated and returned to patients and clinicians, as they were collected as research. Additionally, the genotype array used in the Million Veteran Program does not capture all relevant pharmacogenetic variants, such as *CYP2D6* copy number variations. Therefore, other technological options may be better suited to offer a custom-designed platform for pharmacogenetic testing to veterans at the time a level A drug is prescribed.

### Limitations

Several limitations to the analysis should be noted. The prevalence of pharmacogenetic variants were projected using data from the 1000 Genomes Project rather than directly assayed. There is a dearth of pharmacogenetic information in minority populations, and additional studies are needed in African American and nonwhite Hispanic populations.^36,37,38^ Our analysis was limited to assessment of common variants in the European and African ancestry groups, although our sensitivity analyses showed similar projections when using different populations (eTable 6 in the Supplement). Accounting for veterans of Hispanic ethnicity using the frequencies of pharmacogenetic variants reported for populations from the Americas did not significantly affect our population estimates (eTable 6 in the Supplement); however, it is likely a poor proxy to approximate the prevalence in the diverse Hispanic US population, in which wide variations in admixture of indigenous American, European, African, and Asian ancestry are observed by region of origin.^39,40,41,42^ Given our focus on population-based estimation, we may not have captured the impact of rare variants. Future analyses and clinical implementation efforts will need to account for the changing VHA demographic characteristics over time, and clinical genotyping will need to account for ancestry-specific variants that influence drug response. We did not examine outcomes associated with prescribing level A drugs in veterans with high-risk variants; future studies using data from the Million Veteran Program will be poised to address this limitation. Data on medications were limited to the VHA pharmacy and do not capture prescriptions received in community care settings, nor did we collect data about remote medication use. Additionally, the landscape of medication exposure is rapidly changing, and this analysis reflects the prescription of level A drugs predominantly used at VHA for the study. While we did not report trends over time, we know that the use of warfarin has declined within the VHA as the use of direct-acting oral anticoagulants has increased; however, it is unlikely that warfarin prescribing will be entirely replaced. Our findings changed only slightly when excluding warfarin from analysis, with a 1.6% decrease in the number of level A drug recipients, a 2.3% decrease in the number of new level A drug recipients, and the projected proportion of patients with at least 1 actionable variant changing from 99.4% to 97.6%. As new medications become more popular, revisions will be needed to adjust for the changing prevalence of gene-drug interactions and the addition of new pharmacogenetic tests. We believe that the effect will be greater in the future as we learn more about additional gene-drug interactions. We hope and expect that we will especially learn more about gene-drug interactions relevant to minority populations.^38^ Among current CPIC work in progress, an expansion of opioid guidelines and the release of guidelines on interactions of *CYP2C19* with proton pump inhibitors and *CYP2C9* with celecoxib are likely to affect the veteran population. Given the developing knowledge about pharmacogenetic variants, our estimates are likely to be conservative, and additional veterans may benefit in the future from pharmacogenetic testing beyond those included in this study.

## Conclusions

We estimated that clinically important pharmacogenetic variants are highly prevalent in the VHA population. Almost all veterans would carry an actionable pharmacogenetic variant, and more than half of the population had been exposed to a drug that may be affected by these variants within the 6-year period. These results suggest that preemptive pharmacogenetic testing has the potential to affect pharmacotherapy decisions for most veterans; however, the extent this strategy will reduce the risk of adverse events and minimize therapeutic failures in the veteran population needs to be quantified. These findings do not suggest that pharmacogenetics should replace current strategies for monitoring drug response (eg, international normalized ratio for warfarin) or tailoring therapies (eg, renal or liver function) but rather that integrating pharmacogenetic results within the EHR offers an additional promising avenue to improve outcomes and safety of drugs impacted by these variants.

## Notes

Supplement. **eMethods. **Projections of Pharmacogenetic Variant Prevalence Among VHA Pharmacy Users and Among New Level A Drug Users **eTable 1.** Demographic Characteristics for the Population of Veterans Health Administration Pharmacy Users and Level A Drug Recipients From October 1, 2011, to September 30, 2017 **eTable 2.** Pharmacogenetic Variant Frequencies for Level A Gene-Drug Associations in Reference Population Groups **eTable 3.** Projections for the Prevalence of Actionable Pharmacogenetics Genotypes Among Veterans Health Administration Pharmacy Users **eTable 4.** Projected Frequency of Actionable Phenotypes for *CYP2C9*, *CYP2C19*, and *CYP2D6* Used to Estimate the Proportions of Level A Drug Users With Actionable Phenotypes **eTable 5.** Data Used to Estimate the Admixture of European Ancestry Among African American Veterans Used in Sensitivity Analysis **eTable 6.** Sensitivity Analysis for the Projected Prevalence of Actionable Genotypes Among Veterans Health Administration Pharmacy Users Obtained Under Different Population Models **eTable 7.** Estimation of the Proportion of Veterans Health Administration Pharmacy Users Carrying at Least 1 Pharmacogenetic Variant Allele **eTable 8.** Estimation of the Proportions of Level A Drug Users With Actionable Phenotypes Described in Figure 2 **eTable 9.** Summary of Strong Level A Phenotype-Based Recommendation That the Patient Be Prescribed Alternative or Dose-Adjusted Therapy **eTable 10.** Estimation of the Proportions of Level A Drug Users at Risk of Drug Nonefficacy or Adverse Effects Described in Figure 3 **eFigure.** Result of the Sensitivity Analysis for the Projected Prevalence of Actionable Genotypes Under Different Population Models **eReferences.**
