# Supplementary material for: Projected Prevalence of Actionable Pharmacogenetic Variants and Level A Drugs Prescribed Among US Veterans Health Administration Pharmacy Users
Source: JAMA Netw Open. 2019 Jun 7;2(6):e195345. doi: 10.1001/jamanetworkopen.2019.5345 (PMC6563578; doi:10.1001/jamanetworkopen.2019.5345)
Supplement: Supplement. — eMethods. Projections of Pharmacogenetic Variant Prevalence Among VHA Pharmacy Users and Among New Level A Drug Users eTable 1. Demographic Characteristics for the Population of Veterans Health Administration Pharmacy Users and Level A Drug Recipients From October 1, 2011, to September 30, 2017 eTable 2. Pharmacogenetic Variant Frequencies for Level A Gene-Drug Associations in Reference Population Groups eTable 3. Projections for the Prevalence of Actionable Pharmacogenetics Genotypes Among Veterans Health Administration Pharmacy Users eTable 4. Projected Frequency of Actionable Phenotypes for CYP2C9, CYP2C19, and CYP2D6 Used to Estimate the Proportions of Level A Drug Users With Actionable Phenotypes eTable 5. Data Used to Estimate the Admixture of European Ancestry Among African American Veterans Used in Sensitivity Analysis eTable 6. Sensitivity Analysis for the Projected Prevalence of Actionable Genotypes Among Veterans Health Administration Pharmacy Users Obtained Under Different Population Models eTable 7. Estimation of the Proportion of Veterans Health Administration Pharmacy Users Carrying at Least 1 Pharmacogenetic Variant Allele eTable 8. Estimation of the Proportions of Level A Drug Users With Actionable Phenotypes Described in Figure 2 eTable 9. Summary of Strong Level A Phenotype-Based Recommendation That the Patient Be Prescribed Alternative or Dose-Adjusted Therapy eTable 10. Estimation of the Proportions of Level A Drug Users at Risk of Drug Nonefficacy or Adverse Effects Described in Figure 3 eFigure. Result of the Sensitivity Analysis for the Projected Prevalence of Actionable Genotypes Under Different Population Models eReferences. [file jamanetwopen-2-e195345-s001.pdf]

Supplementary Online Content

Chanfreau-Coffinier C, Hull LE, Lynch JA, et al. Projected prevalence of actionable pharmacogenetic variants and level A drugs prescribed among US Veterans Health Administration pharmacy users. *JAMA Netw Open*. 2019;2(6):e195345. doi:10.1001/jamanetworkopen.2019.5345

eMethods. Projections of Pharmacogenetic Variant Prevalence Among VHA Pharmacy Users and Among New Level A Drug Users

eTable 1. Demographic Characteristics for the Population of Veterans Health Administration Pharmacy Users and Level A Drug Recipients From October 1, 2011 to September 30, 2017

eTable 2. Pharmacogenetic Variant Frequencies for Level A Gene-Drug Associations in Reference Population Groups

eTable 3. Projections for the Prevalence of Actionable Pharmacogenetics Genotypes Among Veterans Health Administration Pharmacy Users

eTable 4. Projected Frequency of Actionable Phenotypes for *CYP2C9*, *CYP2C19*, and *CYP2D6* Used to Estimate the Proportions of Level A Drug Users With Actionable Phenotypes

eTable 5. Data Used to Estimate the Admixture of European Ancestry Among African American Veterans Used in Sensitivity Analysis

eTable 6. Sensitivity Analysis for the Projected Prevalence of Actionable Genotypes Among Veterans Health Administration Pharmacy Users Obtained Under Different Population Models

eTable 7. Estimation of the Proportion of Veterans Health Administration Pharmacy Users Carrying at Least 1 Pharmacogenetic Variant Allele

eTable 8. Estimation of the Proportions of Level A Drug Users With Actionable Phenotypes Described in Figure 2

eTable 9. Summary of Strong Level A Phenotype-Based Recommendation That the Patient Be Prescribed Alternative or Dose-Adjusted Therapy

eTable 10. Estimation of the Proportions of Level A Drug Users at Risk of Drug Nonefficacy or Adverse Effects Described in Figure 3

eFigure. Result of the Sensitivity Analysis for the Projected Prevalence of Actionable Genotypes Under Different Population Models

eReferences.

This supplementary material has been provided by the authors to give readers additional information about their work.

© 2019 Chanfreau-Coffinier C et al. *JAMA Network Open.*

eMethods. Projections of Pharmacogenetic Variant Prevalence Among VHA Pharmacy Users and Among New Level A Drug Users

Estimating the prevalence of actionable PGx variants among VHA Pharmacy users

The prevalence of PGx variants was calculated assuming that the Hardy-Weinberg law applies to the VHA population as a large, randomly mating population with negligible rates of mutation and migration. Under this law, genotype frequencies are expected to follow the frequencies of p^2, $2pq$ and q^2 for p and q being the frequencies of two alleles of a bi-allelic gene. For genes with more than two alleles, we treated variants in the same gene as mutually exclusive, and the frequency of the wild-type allele was calculated as 1 minus the sum of the actionable variant minor allele frequencies (MAFs) reported for a particular racial/ethnic group (eTable 2). This approach is conservative as it ignores existing variants with lower levels of evidence for an abnormal function.

For the gene *G6PD* that is located on the X chromosome, we estimated the frequency of actionable genotypes separately by sex; the frequency of actionable genotypes among male patients (X*Y) was estimated as the sum of MAF, and as the frequency of homozygote carriers for female patients (X*X*); we weighted separately the gender frequencies for the two ancestry groups to account for the greater number of women Veterans of African ancestry vs European ancestry (16% vs 8%, respectively) (eTable 3).

Estimations using weighted phenotype frequencies allowed us to account for the frequent combinations of genetic variants at those three loci, and the variations in number of copies for *CYP2D6*.[1]

Modeling the population diversity

To account for the diversity of the VHA population we weighted those estimates to produce the number of actionable genotypes among VHA patients with a representation of 15% patients of African ancestry and 85% of patients of European ancestry that are the two predominant groups, and reflected the proportions of VHA Pharmacy users of African ancestry in our sample (eTable 1).[2,3] VHA enrollees with a race/ethnicity that was either unknown or Hispanic, were merged into the European ancestry group.[2,3] Additionally, we performed sensitivity analyses to model the population diversity, accounting for European admixture among African Americans (eTables 5 & 6).

Estimating the proportion of VHA Pharmacy users who would carry at least one actionable variant

We estimated the proportion of Veterans who would carry at least one actionable variant as 1 minus the product of the probabilities of a wildtype genotype at each locus (compiled in eTable 2, as 1 minus the sum of frequencies for the Level-A variant alleles). Probabilities were treated as independent as all genes included in the study are carried by separate chromosomes, except for *CYP2C9* and *CYP2C19* located both on chromosome 10. A sensitivity analysis was performed accounting for the linkage of the variant alleles of *CYP2C9* with the wildtype allele of *CYP2C19* genes which yielded similar estimates.

Identification of patients receiving a new prescription for clopidogrel within 30 days after a percutaneous coronary intervention

In the case of clopidogrel, clinical guidelines are strongest for the impact of PGx testing in the setting of percutaneous coronary intervention (PCI); therefore, we reported the projected number of patients with actionable phenotypes among those patients receiving a new prescription for clopidogrel within 30 days after a PCI, as indicated by the presence of a procedure code [CPT 92928, 92929, 92933, 92934, 92937, 92938, 92941, 92943, 92944, 92980, 92981, C1874-C1877, C9600-C9603].[4] As many Veterans undergoing PCI have the procedure done at a non-VA medical center, we included patients with a procedure code either from the OMOP Procedure Occurrence table for all procedures performed at VA, and from the CDW Fee Basis table for all procedures performed outside VA and paid by VA, which indicates that PCI was performed in the community.

Projecting the clinical impact of Level-A gene-drug interactions

Using the phenotype data, we identified a subgroup of patients with projected phenotypes putting them at high risk of being exposed to a drug for which they have a high likelihood of 1) non-efficacy and/or 2) drug toxicity and adverse events, and characterized the anticipated nature of the toxicities. We limited our analyses to the medications in Figure 1 with a strong CPIC level-A phenotype-based recommendation that the patient be prescribed alternative or dose-adjusted therapy. For the drug warfarin, we accounted for the combinations of alleles between

© 2019 Chanfreau-Coffinier C et al. *JAMA Network Open.*

the two genes assuming independence of genes carried on different chromosomes (*CYP2C9* on chromosome 10 *and VKORC1* on chromosome 16).These recommendations applied to the following drug-gene interactions: simvastatin-*SLCO1B1* "intermediate to low function" carriers; codeine-*CYP2D6* ultra-rapid and poor metabolizers; clopidogrel-*CYP2C19* poor metabolizers; allopurinol-*HLA-B*5801* carriers; paroxetine-*CYP2D6* ultra-rapid metabolizers. We graphically depicted the absolute number of patients who were exposed to these high-risk medications by their risk of drug non-efficacy and/or toxicity.

© 2019 Chanfreau-Coffinier C et al. *JAMA Network Open.*

eTable 1. Demographic Characteristics for the Population of Veterans Health Administration Pharmacy Users And Level A Drug Recipients From October 1, 2011 to September 30, 2017

	**No. (%) of Patients**					
**Characteristic**	**Pharmacy users** [a]		**Level-A drug users** [b]		**New Level-A drug users** [c]	
Total, No.	7,769,359		4,259,153		2,943,872	
Age at FY2012, mean (SD), y	58.1 (17.8)		60.2 (16.2)		57.1 (16.3)	
Sex						
Men	7,021,504	(90.4%)	3,926,132	(92.2%)	2,668,941	(90.7%)
Women	747,564	(9.6%)	332,929	(7.8%)	274,859	(9.3%)
Not specified	291	(<0.1%)	92	(<0.1%)	72	(<0.1%)
Race/Ethnicity						
African American	1,195,906	(15.4%)	703,837	(16.5%)	535,992	(18.2%)
White	5,153,274	(66.3%)	2,929,081	(68.8%)	1,984,045	(67.4%)
Hispanic	450,692	(5.8%)	251,422	(5.9%)	192,328	(6.5%)
Other [d]	187,000	(2.4%)	96,276	(2.3%)	71,410	(2.4%)
Unknown	782,487	(10.1%)	278,537	(6.5%)	160,097	(5.4%)

Abbreviations: FY, fiscal year; VHA, Veterans Health Administration

[a] Unique patients identified in the Veterans Affairs Corporate Data Warehouse based at least one prescription received from VHA Pharmacy in fiscal years 2012-2017

[b] Patients with at least one prescription for a Level-A drug received from VHA Pharmacy in fiscal years 2012-2017.

[c] Patients with a new prescription for a Level-A drug received from VHA Pharmacy in fiscal years 2012-2017.

[d] Other race: Asian, American Indian or Alaskan Native, and Native Hawaiian or Pacific Islander

© 2019 Chanfreau-Coffinier C et al. *JAMA Network Open.*

eTable 2. Pharmacogenetic Variant Frequencies for Level A Gene-Drug Associations in Reference Population Groups

					Population-specific variant frequency[a]			
**Gene**	**Allele**	**Functional status**	**Carrier affected[b]**	**Reference SNP**	**AFR**	**EUR**	**ASW**	**AMR**
CYP2C9	*2	decreased function	1 or 2 copies	rs1799853	0.008	0.124	0.041	0.099
CYP2C9	*3	decreased function	1 or 2 copies	rs1057910	0.002	0.073	0.037	0.016
CYP2C9	*5	possible decreased	1 or 2 copies	rs28371686	0.017	-	0.025	0.001
CYP2C9	*6	no function	1 or 2 copies	rs9332131	0.008	-	-	-
CYP2C9	*8	possible decreased	1 or 2 copies	rs7900194	0.053	0.002	0.033	0.001
CYP2C9	*11	possible decreased	1 or 2 copies	rs28371685	0.024	0.002	0.008	0.001
VKORC1	1639 G>A	increased warfarin sensitivity	1 or 2 copies	rs9923231	0.100	0.410	0.148	0.411
CYP2C19	*2	No function	2 copies	rs12767583	0.169	0.124	0.139	0.104
CYP2C19	*3	No function	2 copies	rs4986893	0.002	0.073	-	-
CYP2C19	*4	No function	2 copies	rs28399504	-	-	-	0.003
CYP2C19	*8	No function	2 copies	rs41291556	0.001	-	0.008	-
CYP2C19	*17	Increased function	1 or 2 copies	rs12248560	0.235	0.224	0.197	0.120
CYP2D6	*3	No function	2 copies	rs35742686	0.002	0.019	0.016	0.006
CYP2D6	*4	No function	2 copies	rs3892097	0.061	0.186	0.123	0.130
CYP2D6[5]	*5	No function	2 copies	n/a; deletion	0.061	0.028	0.064	0.021
CYP2D6	*6	No function	2 copies	rs5030655	0.001	0.020	0.008	0.003
CYP2D6	*7	No function	2 copies	rs5030867	-	0.000	-	-
CYP2D6	*8	No function	2 copies	rs5030865	-	0.000	-	-
CYP2D6	*9	decreased function	2 copies	rs5030656	0.001	0.026	0.008	0.013
CYP2D6	*10	decreased function	2 copies	rs1065852	0.041	0.028	0.156	0.148
CYP2D6	*17	decreased function	2 copies	rs28371706	0.218	0.020	0.148	0.009
CYP2D6	*29	decreased function	2 copies	rs59421388	0.065	0.001	0.041	0.003
CYP2D6	*41	decreased function	2 copies	rs28371725	0.087	0.087	0.016	0.062
CYP2D6[6]	Gene duplication	Increased function	> 1 copy	n/a	0.045	0.033	0.034	0.048
CYP3A5[7]	*1	functional allele	1 or 2 copies	n/a	0.560	0.078	0.605	0.202

© 2019 Chanfreau-Coffinier C et al. *JAMA Network Open.*

					Population-specific variant frequency[a]			
**Gene**	**Allele**	**Functional status**	**Carrier affected[b]**	**Reference SNP**	**AFR**	**EUR**	**ASW**	**AMR**
SLCO1B1	*5	decreased function	1 or 2 copies	rs4149056	0.014	0.161	0.066	0.134
UGT1A1	*80	decreased function	2 copies	rs887829	0.493	0.298	0.459	0.379
TPMT	*2	No function	1 or 2 copies	rs1800462	0.001	0.006	0.008	0.006
TPMT	*3	No function	1 or 2 copies	rs1800460	0.003	0.028	0.025	0.040
DPYD	*2A	No function	1 or 2 copies	rs3918290	0.001	0.005	0.008	0.001
DPYD	D949V	decreased function	1 or 2 copies	rs67376798	0.001	0.007	0.008	0.003
G6PD[c]	A- [202A;376G]	deficient	Male: 1 copy; female 2 copies	rs1050828, rs1050829	0.134	-	0.167	0.013
G6PD[c]	Asahi [202A; 376A]	deficient	Male: 1 copy; female 2 copies	rs1050828	0.001	-	-	-
G6PD[c]	A [202G;376G]	deficient	Male: 1 copy; female 2 copies	rs1050829	0.204	0.004	0.125	0.015
IFNL3B	r151-2G>A	decreased response	1 or 2 copies	rs12979860	0.390	0.630	0.320	0.601
HLA-A[8]	*31:01	hypersensitivity reaction	1 or 2 copies	n/a	0.005	0.028	0.010	0.064
HLA-B[8]	*57:01	hypersensitivity reaction	1 or 2 copies	n/a	0.008	0.032	0.001	0.016
HLA-B[8]	*58:01	severe cutaneous adverse reactions	1 or 2 copies	n/a	0.054	0.013	0.039	0.011
HLA-B[8]	*15:02	Stevens-Johnson syndrome, toxic epidermal necrolysis	1 or 2 copies	n/a	0.0001	0.0004	0.001	0.0004

Abbreviation: AFR: African ancestry; AMR: Americas; ASW, People with African Ancestry in Southwest USA; EUR, European ancestry; SNP, single nucleotide polymorphism.

[a] Variant frequency reported as the minor allele frequency for the three populations AFR, EUR and AMER, and the ASW group in the 1000 Genomes Project Phase 3[9], else otherwise indicated by a citation. SNP are specific each for a gene variant, except in the case of *G6PD* deficient alleles characterized by the combination of two SNPs. Variants of a same gene are considered mutually exclusive.

[b] Specifies if carriers affected by the gene-drug association are either homozygous (i.e., 2 copies), or include both homozygous and heterozygous carriers (i.e., 1 or 2 copies)

[c] Frequencies of *G6PD* deficient alleles in the table take in account the linkage disequilibrium observed between rs1050828 and rs1050829 (estimated using LD pair).[10]

© 2019 Chanfreau-Coffinier C et al. *JAMA Network Open.*

eTable 3. Projections for the Prevalence of Actionable Pharmacogentics Genotypes Among Veterans Health Administration Pharmacy Users

		AFR subpopulation					EUR subpopulation					VHA pop
**Gene**	**Alleles, grouped by function**	**Allele freq.**	**Heterozygote freq. [2*p*wt]**	**Compound heterozygote freq. [2*p*r]**	**Homozygote freq. [p*p]**	**Actionable GT**	**Allele freq.**	**Heterozygote freq. [2*p*wt]**	**Compound heterozygote freq. [2*p*r]**	**Homozygote freq. [p*p]**	**Actionable GT**	**Actionable GT**
CYP 2C9	wt (other than *2-*11)	0.888					0.799					
	*2	0.008	0.014	0.002	<0.001		0.124	0.198	0.019	0.015		
	*3	0.002	0.004	<0.001	<0.001		0.073	0.117	0.001	0.005		
	*5	0.017	0.030	0.003	<0.001		-	<0.001	-	-		
	*6	0.008	0.014	0.001	<0.001		-	<0.001	-	-		
	*8	0.053	0.094	0.003	0.003		0.002	0.003	<0.001	<0.001		
	*11	0.024	0.043		0.001		0.002	0.003		<0.001		
	**Decreased function (1 or 2 copies)**		0.199	0.009	0.004	**21.1%**		0.321	0.020	0.021	**36.2%**	**33.9%**
VKORC1	wildtype	0.900					0.59					
	(-1639 G>A)	0.100	0.180	n/a	0.010		0.41	0.484	n/a	0.168		
	**Increased sensitivity (1 or 2 copies)**		0.180	n/a	0.010	**19.0%**		0.484	n/a	0.168	**65.2%**	**58.3%**
CYP 2C19	wildtype (other than *2-*17)	0.593					0.579					
	*2	0.169	0.200	0.001	0.029		0.124	0.144	0.018	0.015		
	*3	0.002	0.002	<0.001	<0.001		0.073	0.085	-	0.005		
	*4	-	-	-	-		-	-	-	-		
	*8	0.001	0.001		<0.001		-	-		-		
	**Decreased function (2 copies)**		0.204	0.001	0.029	**23.4%**		0.228	0.018	0.021	**26.7%**	**26.2%**
	*17	0.235	0.279	0.114	0.055		0.224	0.260	0.118	0.050		
	**Increased function (1 or 2 copies)**		0.279	0.114	0.055	**44.8%**		0.260	0.118	0.050	**42.8%**	**43.1%**

© 2019 Chanfreau-Coffinier C et al. *JAMA Network Open.*

		AFR subpopulation					EUR subpopulation					VHA pop
**Gene**	**Alleles, grouped by function**	**Allele freq.**	**Heterozygote freq. [2*p*wt]**	**Compound heterozygote freq. [2*p*r]**	**Homozygote freq. [p*p]**	**Actionable GT**	**Allele freq.**	**Heterozygote freq. [2*p*wt]**	**Compound heterozygote freq. [2*p*r]**	**Homozygote freq. [p*p]**	**Actionable GT**	**Actionable GT**
	**Total actionable for CYP2C19**					**68.1%**					**69.5%**	**69.3%**
CYP 2D6	wildtype (all other than *3-*41 or duplicate)	0.463					0.585					
	*3	0.002	0.002	0.002	<0.001		0.019	0.022	0.015	<0.001		
	*4	0.061	0.056	0.058	0.004		0.186	0.218	0.078	0.035		
	*5	0.061	0.056	0.050	0.004		0.028	0.033	0.010	<0.001		
	*6	0.001	0.001	<0.001	<0.001		0.020	0.023	0.006	<0.001		
	*7	-	-	-	-		-	-	-	-		
	*8	-	-	-	-		-	-	-	-		
	*9	0.001	0.001	<0.001	<0.001		0.026	0.030	0.007	<0.001		
	*10	0.041	0.038	0.030	0.002		0.028	0.033	0.006	<0.001		
	*17	0.218	0.202	0.066	0.048		0.02	0.023	0.003	<0.001		
	*29	0.065	0.060	0.011	0.004		0.001	0.001	<0.001	<0.001		
	*41	0.087	0.081				0.087	0.102				
	**Decreased function (2 copies)**		n/a	n/a	0.061	**6.1%**		n/a	n/a	0.038	**3.8%**	**4.1%**
	**Increased function (frequency of phenotype)**	n/a				**4.5%**	n/a				**3.3%**	**3.4%**
	**Total actionable for CYP2D6**					**10.5%**					**7.1%**	**7.6%**
CYP 3A5	alleles other than *1 [a]	0.440					0.922					
	*1	0.560	0.493		0.314		0.078	0.144		0.006		
	**Extensive metabolizer (1 or 2 copies)**		0.493		0.314	**80.6%**		0.144		0.006	**15.0%**	**24.8%**

© 2019 Chanfreau-Coffinier C et al. *JAMA Network Open.*

		AFR subpopulation					EUR subpopulation					VHA pop
**Gene**	**Alleles, grouped by function**	**Allele freq.**	**Heterozygote freq. [2*p*wt]**	**Compound heterozygote freq. [2*p*r]**	**Homozygote freq. [p*p]**	**Actionable GT**	**Allele freq.**	**Heterozygote freq. [2*p*wt]**	**Compound heterozygote freq. [2*p*r]**	**Homozygote freq. [p*p]**	**Actionable GT**	**Actionable GT**
SLCO1B1	wildtype (other than *5)	0.986					0.839					
	*5	0.014	0.028		<0.001		0.161	0.270		0.0260		
	**Intermediate to low function (1 or 2 copies)**		0.028		<0.001	**2.8%**		0.270		0.0260	**29.6%**	**25.6%**
UGT1A1	wildtype (other than *80)	0.507					0.702					
	*80	0.493	0.500		0.243		0.298	0.418		0.089		
	**Deficiency (2 copies)**		n/a		0.243	**24.3%**		n/a		0.089	**8.9%**	**11.2%**
TPMT	wildtype (other than *2,*3)	0.996					0.966					
	*2	0.001	0.002	<0.001	<0.001		0.006	0.012	<0.001	<0.001		
	*3	0.003	0.006	<0.001	<0.001		0.028	0.054		<0.001		
	**Deficiency (1 or 2 copies)**		0.008	<0.001	<0.001	**0.8%**		0.066	<0.001	<0.001	**6.7%**	**5.8%**
DPYD	wildtype (other than *2A, D949V)	0.998					0.988					
	*2A	0.001	0.002	<0.001	<0.001		0.005	0.010	<0.001	<0.001		
	D949V	0.001	<0.001		<0.001		0.007	<0.001		<0.001		
	**Deficiency (1 or 2 copies)**		0.002	<0.001	<0.001	**0.2%**		<0.001	<0.001	<0.001	**1.0%**	**0.9%**
G6PD	wildtype [202G;376A]	0.661					0.100					
	A- [202A;376G]	0.134					0					
	Asahi [202A; 376A]	0.001					0					

© 2019 Chanfreau-Coffinier C et al. *JAMA Network Open.*

		AFR subpopulation					EUR subpopulation					VHA pop
**Gene**	**Alleles, grouped by function**	**Allele freq.**	**Heterozygote freq. [2*p*wt]**	**Compound heterozygote freq. [2*p*r]**	**Homozygote freq. [p*p]**	**Actionable GT**	**Allele freq.**	**Heterozygote freq. [2*p*wt]**	**Compound heterozygote freq. [2*p*r]**	**Homozygote freq. [p*p]**	**Actionable GT**	**Actionable GT**
G6PD	A [202G;376G]	0.204					0.004					
	**Deficiency - all**	0.339					0.004					
	Male (XY)		0.339		n/a			0.004		n/a		
	Female (XX)		n/a		0.115			n/a		<0.001		
	weighted for gender		0.284		0.018	**30.3%**		0.004		<0.001	**0.4%**	**4.9%**
IFNL3	other than r151-2G>A	0.610					0.370					
	r151-2G>A	0.390	0.476		0.152		0.630	0.466		0.400		
	**Unfavorable response**		0.476		0.152	**62.8%**		0.466		0.400	**86.3%**	**82.8%**
HLA	**Presence (1 or 2 copies)**											
HLA-A	*31:01	0.005	0.010		<0.001	**1.0%**	0.028	0.054		0.001	**5.5%**	**4.8%**
HLA-B	*57:01	0.008	0.016		<0.001	**1.6%**	0.032	0.062		0.001	**6.3%**	**5.6%**
HLA-B	*58:01	0.054	0.102		0.003	**10.5%**	0.013	0.026		<0.001	**2.6%**	**3.8%**
HLA-B	*15:02	0.0001	<0.001		<0.001	**0.0%**	<0.001	0.001		<0.001	**0.1%**	**0.1%**

Abbreviation: Freq.: frequency; GT: genotype; pop: population

Population-specific prevalence of actionable genotypes were weighted to generate population estimates using the weights of 15% for AFR and 85% for EUR (Model 1 in eTable 5)

We assume that the variants were mutually exclusive, and the frequency of wildtype alleles is obtained as 1 minus the sum of frequencies of the variant alleles.

The frequency of homozygote genotypes is calculated as the square of the frequency of the variant (1 copy on each chromosome), and the frequency of heterozygote is estimated as 2 times the product of the variant allele by the other allele. The frequencies of compound heterozygotes combining two variant alleles were calculated in series to avoid double-counting of combinations, e.g. allele A was combined to alleles B to E, then allele B is combined to allele C to E, allele C is combined to allele D to e, and so on.

[a] In the case of *CYP3A5*, the actionable allele is the reference allele *1 that encodes functional CYP3A5 and for which dose adjustment is recommended; other alleles are nonfunctional.

© 2019 Chanfreau-Coffinier C et al. *JAMA Network Open.*

eTable 4. Projected Frequency of Actionable Phenotypes for *CYP2C9*, *CYP2C19*, and *CYP2D6* Used to Estimate the Proportions of Level A Drug Users With Actionable Phenotypes

Gene	Phenotype	Prevalence for AFR	Prevalence for EUR	Projected for VHA population
CYP2C9[11]	Normal metabolizer	75.2%	64.0%	65.7%
	Intermediate metabolizer	23.1%	32.0%	30.7%
	Poor metabolizer	1.8%	4.0%	3.7%
CYP2C19[12]	Ultrarapid Metabolizer	2.3%	4.6%	4.3%
	Rapid Metabolizer	13.6%	26.9%	24.9%
	Normal Metabolizer	16.8%	39.2%	35.8%
	Intermediate Metabolizer	24.1%	26.8%	26.4%
	Poor Metabolizer	4.8%	2.5%	2.9%
	Unknown	*38.4%*	*0.0%*	*5.8%*
CYP2D6[6]	Ultrarapid Metabolizer	4.5%	3.3%	3.4%
	Normal Metabolizer	71.9%	74.9%	74.5%
	Normal Metabolizer, Ultrarapid Metabolizer	0.8%	1.1%	1.1%
	Intermediate Metabolizer	12.6%	7.2%	8.0%
	Poor Metabolizer	1.9%	6.1%	5.4%
	Unknown	8.4%	7.4%	7.5%

Abbreviations: AFR: African ancestry; EUR, European ancestry

© 2019 Chanfreau-Coffinier C et al. *JAMA Network Open.*

eTable 5. Data Used to Estimate the Admixture of European Ancestry Among African American Veterans Used in Sensitivity Analysis

References	Methods	Genotyped population (Study)	Sample size	EUR ancestry
Parra 1998[13]	PCR on 9 loci	10 US sites	1020	16.4%
Reiner 2005[14]	22 SNPs	3 US sites (CHS study)	810	20.1%
Yaeger 2008[15]	107 SNPs	New York region	50	14.7%
Allison 2010[16]	97 SNPs	6 US regions (MESA study)	712	20.2%
Murray 2010[17]	416 SNPs	Baltimore/DC (GRAAD study)	906	19.7%
Bryc 2015[18]	>500,000 SNPs	US national (23andMe)	1970	24.0%
Banda 2015[19]	250,000 SNPs	Northern California (Kaiser)	3365	26.0%
Baharian 2016[20]	>500,000 SNPs	South West (SCCS)	2128	14.0%
Baharian 2016[20]	>500,000 SNPs	National (HRS National)	1501	16.7%
Baharian 2016[20]	>500,000 SNPs	South West (ASW 1000G)	97	21.3%
Mathias 2016[21]	>500,000 SNPs	8 US sites	328	18.8%
Weighted average			12887	20.5%

Abbreviations: ASW, People with African Ancestry in Southwest USA; CHS, Cardiovascular Health Study; EUR, European ancestry; GRAAD, Genomic Research on Asthma in the African Diaspora; HRS, Health and Retirement Study; MESA, Multi-Ethnic Study of Atherosclerosis; PCR, polymerase chain reaction; SCCS, Southern Community Cohort Study; SNP, single nucleotide polymorphism;

Number of ancestry markers analyzed, the number of sites and geographic distribution of the genotyped population, contribution of European ancestry among African American participants, and average value weighted for the sample sizes.
Values reported are specific for African American participants in each study

© 2019 Chanfreau-Coffinier C et al. *JAMA Network Open.*

eTable 6. Sensitivity Analysis for the Projected Prevalence of Actionable Genotypes Among Veterans Health Administration Pharmacy Users Obtained Under Different Population Models

	**Population Estimates for the prevalence of PGx variants**							
**Gene**	**Model 1** AFR 15%	**Model 2** AFR 20%	**Model 3** AFR 15% 21% EUR admix	**Model 4** AFR 20% 21% EUR admix	**Model 5** ASW 15%	**Model 6** AFR 15% AMR 7%	**Average value with 95% CI**	
CYP2C9	33.9%	33.2%	33.3%	32.4%	34.7%	32.9%	33.4%	± 0.6%
VKORC1	58.3%	56.0%	58.2%	55.9%	59.5%	58.3%	57.7%	± 1.1%
CYP2C19 Decreased function	26.2%	26.0%	25.4%	24.9%	25.9%	25.6%	25.7%	± 0.3%
CYP2C19 Increased function	43.1%	43.2%	41.8%	41.5%	42.3%	41.9%	42.3%	± 0.5%
CYP2D6 Decreased function	4.1%	4.3%	3.8%	3.8%	4.2%	4.2%	4.1%	± 0.1%
CYP2D6 Increased function	3.4%	3.5%	3.2%	3.2%	3.3%	3.6%	3.4%	± 0.1%
CYP3A5	24.8%	28.1%	21.0%	23.0%	25.4%	26.3%	24.8%	± 1.8%
SLCO1B1	25.6%	24.2%	25.7%	24.3%	27.1%	25.3%	25.4%	± 0.8%
UGT1A1	11.2%	12.0%	10.0%	10.4%	10.7%	11.6%	11.0%	± 0.5%
TPMT	5.8%	5.5%	5.8%	5.5%	6.7%	6.0%	5.9%	± 0.3%
DPYD	0.9%	0.8%	0.9%	0.8%	1.1%	0.8%	0.9%	± 0.1%
G6PD	4.9%	6.4%	3.2%	4.1%	4.2%	5.0%	4.6%	± 0.8%
IFNL3	82.8%	81.6%	82.5%	81.2%	81.4%	82.6%	82.0%	± 0.5%
*HLA-A*31:01*	4.8%	4.6%	4.8%	4.6%	5.0%	5.3%	4.9%	± 0.2%
*HLA-B*57:01*	5.6%	5.4%	5.5%	5.3%	5.4%	5.4%	5.4%	± 0.1%
*HLA-B*58:01*	3.8%	4.2%	3.2%	3.4%	3.3%	3.7%	3.6%	± 0.3%
*HLA-B*15:02*	0.1%	0.1%	0.1%	0.1%	0.1%	0.1%	0.1%	± 0.0%

Abbreviations: AFR: African ancestry; AMR: Americas; ASW, People with African Ancestry in Southwest USA; EUR, European ancestry

Model 1 used in the primary analysis accounting for a mix of 15% patients of African ancestry and 85% of patients of European ancestry. Model 2, alternative weights for 20% patients of African ancestry and 80% of patients of European ancestry. Models 3 and 4 models the admixture of African American patients with a 21% contribution of European ancestry; the

© 2019 Chanfreau-Coffinier C et al. *JAMA Network Open.*

21% contribution was estimated based on the literature review summarized in eTable4. Model 5 applies the variant frequencies for the African American in Southwest USA in the 1000 genome project with weights of 15% ASW/85% EUR. Model 6 tests the effect of accounting the contribution of populations from American origin, and models the population as 15% for AFR, 7% AMR (population from Americas), and 78% EUR.

PGx variants values for each population are listed in eTable 2; all calculations follow the same model as applied to Model 1 in eTable 1. Average values for the six models with the range of 95% CI interval are presented in the right-hand column.

For gender adjustment in *G6PD* estimation, we applied proportions of women patients for each population as reported in the 2017: AFR and ASW, 16% women patients; AMR, 11%; and EUR, 8%.

© 2019 Chanfreau-Coffinier C et al. *JAMA Network Open.*

eTable 7. Estimation of the Proportion of Veterans Health Administration Pharmacy Users Carrying at Least 1 Pharmacogenetic Variant Allele

Gene	Genotypes with wildtype phenotype	Prevalence for AFR	Prevalence for EUR
CYP2C9	wt/wt	0.789	0.638
VKORC1	wt/wt	0.810	0.348
CYP2C19	total	0.556	0.563
	wt/wt	*0.352*	*0.335*
	wt/var with decreased function	*0.204*	*0.228*
CYP2D6	total	0.712	0.828
	wt/wt	*0.214*	*0.342*
	wt/var with decreased function	*0.497*	*0.486*
CYP3A5[a]	(other than *1)/(other than *1)	0.194	0.850
SLCO1B1	wt/wt	0.972	0.704
	total	0.757	0.911
	wt/wt	*0.257*	*0.493*
	*wt/*80*	*0.500*	*0.418*
TPMT	wt/wt	0.992	0.933
DPYD	wt/wt	0.996	0.976
G6PD	1 wt copy (male)	0.661	0.911
IFNL3	wt/wt	0.372	0.137
HLA-A	wt/wt	0.990	0.945
HLA-B	wt/wt	0.938	0.955
	Product of all probabilities per group	**0.008**	**0.006**
	Weighted probability of all wildtype	P	**0.006**
	Probability of at least one actionable variant	1- P	**0.994**

Abbreviations: AFR: African ancestry; EUR, European ancestry; wt, wildtype

The probability of having at least one actionable variant was calculated as 1 minus the probability of a wildtype phenotype at each of the loci analyzed, which is the product of the probabilities of a wildtype phenotype for each gene. For *CYP2C19*, *CYP2D6* and *SLCO1B1*, a wildtype phenotype can result from carrying 1 or 2 copies of the wildtype allele, and the probability of a wildtype phenotype is the sum of the probabilities of the two genotypes that are mutually exclusive.

[a] For *CYP3A5*, *1 is the actionable allele, and combinations of other alleles results in a non-actionable phenotype.

The model assumes that the genes are independent from each other as they are carried by different chromosomes, except for CYP2C9 and CYP2C19 that are both on chromosome 10. We performed a sensitivity analysis to test the impact of linkage disequilibrium between *CYP2C9* and *CYP2C19* that results in a complete linkage of *CYP2C9* wildtype allele with *CYP2C19* variant alleles. The frequency of double allele *CYP2C9wt;CYP2C19wt* is then the frequency of *CYP2C9wt* minus the frequency of *CYP2C9wt;CYP2C19var* (i.e., the frequency of *CYP2C19* variant because of complete linkage). After adjustment for linkage disequilibrium, the results were unchanged: the prevalence of *CYP2C9wt* alleles were 0.481 for AFR and 0.378 for EUR, and the frequencies of a wildtype phenotype for *CYP2C9* were 0.231 and 0.142. The final result in the table above was 0.995.

© 2019 Chanfreau-Coffinier C et al. *JAMA Network Open.*

As warfarin was associated to a high percentage of actionable variants but the number of warfarin prescriptions are on the decline, we performed an additional sensitivity analysis without including the genes associated to warfarin, *CYP2C9* and *VKORC1*. The product of the probabilities of a wildtype phenotype for the ten other genes listed in the table resulted in a decrease of the probability of carrying at least one actionable variant from 0.994 to 0.976.

© 2019 Chanfreau-Coffinier C et al. *JAMA Network Open.*

eTable 8. Estimation of the Proportions of Level A Drug Users With Actionable Phenotypes Described in Figure 2

			Actionable phenotypes	
**Drug**	**Unique new users (No.)**	**Associated genes**	**Total prevalence**	**Projected number of users (No.)**
Tramadol	923,671	*CYP2D6* [b]	8.9%	82,092
Ondansetron	604,226	*CYP2D6* [c]	3.5%	20,816
Simvastatin	533,928	*SLCO1B1* [a]	25.6%	136,599
Simvastatin users prescribed 80 mg initial dose	125,119	*SLCO1B1* [a]	25.6%	32,010
Codeine	528,159	*CYP2D6* [b]	8.9%	46,941
Clopidogrel	338,295	*CYP2C19* [f]	29.2%	98,900
Clopidogrel PCI patients[e]	51,094	*CYP2C19* [f]	29.2%	14,937
Citalopram	266,952	*CYP2C19* [d]	7.2%	19,100
Allopurinol	215,055	*HLA-B:58*01*	3.8%	8,172
Warfarin	205,177	*VKORC1, CYP2C9* [g]	72.6%	148,928
Amitriptyline	174,693	*CYP2C19, CYP2D6* [h]	40.8%	71,216
Escitalopram	170,690	*CYP2D6* [b]	8.9%	15,170

Abbreviation: PCI, percutaneous coronary intervention
[a] intermediate to low function
[b] ultra-rapid metabolizers and poor metabolizers
[c] ultra-rapid metabolizers only
[d] ultra-rapid metabolizers, and poor metabolizers
[e] new users of clopidogrel receiving the drug within 30 days after a percutaneous coronary intervention
[f] intermediates and poor metabolizers
[g] poor/intermediate metabolizer for *CYP2C9* and/or increased sensitivity linked to *VKORC1*, calculated as the frequency of *CYP2C9 poor/intermediate metabolizer;VKORC1 sensitive*, plus *CYP2C9 poor/intermediate metabolizer;VKORC1 wildtype* plus *CYP2C9 wildtype;VKORC1 sensitive*
[h] *CYP2D6* (ultra, intermediate or poor metabolizer), plus *CYP2C19* (ultra, rapid or poor metabolizer) combined with normal *CYP2D6*

Population prevalence estimates from eTable2 for *SLCO1B1, HLA-B:58*01* and *VKORC1;* and from eTable 4 for *CYP2C9, CYP2C19* and *CYP2D6.*

© 2019 Chanfreau-Coffinier C et al. *JAMA Network Open.*

eTable 9. Summary of Strong Level A Phenotype-Based Recommendation That the Patient Be Prescribed Alternative or Dose-Adjusted Therapy

**Drug**	**Gene**	**Phenotype**	**CPIC Recommendation**
Allopurinol[22]	*HLA-B*58:01*	Presence of the variant	Significantly **increased risk of allopurinol-induced severe cutaneous adverse reaction** - allopurinol contraindicated (Strong)
Clopidogrel [12] (PCI)	*CYP2C19*	Poor metabolizer	Select alternative antiplatelet agent; Increased risk of **non-efficacy and adverse CV events** (Strong)
Codeine[6]	*CYP2D6*	Ultra-rapid metabolizer	Avoid codeine use due to **potential for toxicity** (Strong)<br>Considerations for alternative opioids - Alternatives that are not affected by this *CYP2D6* phenotype include morphine and non-opioid analgesics. Tramadol and, to a lesser extent, hydrocodone and oxycodone, are not good alternatives because their metabolism is affected by *CYP2D6* activity.
	CYP2D6	Poor Metabolizer	Avoid codeine use due to **lack of efficacy** (Strong)<br>Considerations for alternative opioids - Alternatives that are not affected by this *CYP2D6* phenotype include morphine and non-opioid analgesics. Tramadol and, to a lesser extent, hydrocodone and oxycodone, are not good alternatives because their metabolism is affected by *CYP2D6* activity.
Simvastatin[23]	*SLCO1B1*	Intermediate function	Prescribe a lower dose or consider an alternative statin due to **increased myopathy risk** (strong) - intermediate myopathy risk.
	SLCO1B1	Low function	Prescribe a lower dose or consider an alternative statin -**increased myopathy risk** (strong) - high myopathy risk.
Warfarin[11]	*VKORC1*	Increased sensitivity	For patients who self-identify as non-African ancestry, strong data to support using a *CYP2C9*/*VKORC1* pharmacogenetic algorithm **to guide warfarin dosing**.
	CYP2C9	Poor metabolizer	

Medications with a strong CPIC level A recommendation to either avoid or dose-adjust a medication based on available pharmacogenetic test results are included.

© 2019 Chanfreau-Coffinier C et al. *JAMA Network Open.*

eTable 10. Estimation of the Proportions of Level A Drug Users at Risk of Drug Nonefficacy or Adverse Effects Described in Figure 3

Drug	Gene	Phenotype	Unique drug users (No.)	Projected w/ phenotype (%)[a]	Projected w/ phenotype (No.)	Total of actionable phenotypes by drug (No.)[b]	Proportion of all actionable variants by drug (%)
Allopurinol	*HLA-B*58:01*	Presence of the variant	215,055	3.8%	8,172	8,172	100.0%
Clopidogrel PCI patients	*CYP2C19*	Poor metabolizer	51,094	2.9%	1,482	14,937	9.9%
Codeine	*CYP2D6*	Ultra-rapid metabolizer	528,159	3.5%	18,486	46,941	39.4%
	CYP2D6	Poor Metabolizer	528,159	5.4%	28,521	46,941	60.8%
Simvastatin	*SLCO1B1*	Intermediate function	533,928	23.4%	124,939	136,599	91.5%
	SLCO1B1	Low function	533,928	2.2%	11,746	136,599	8.6%
Warfarin[c]	*VKORC1*	Increased sensitivity	174,400[c] (with projected European ancestry)	66.6%[c]	116,151	148,928 (all ancestries)	78.0%
	CYP2C9	Poor metabolizer					

Abbreviations: PCI: percutaneous coronary intervention

Medications with a strong CPIC level A recommendation to either avoid or dose-adjust a medication based on available pharmacogenetic test results, genes and phenotypes associated with an increased risk of toxicity and/or adverse drug reaction in response to drug exposure, number of unique drug users for the drug in 2012-2017, projected prevalence and number of patients with specific phenotype at a high risk of adverse drug reaction and/or non-efficacy, total number of patients with an actionable phenotype

[a]The percentage represents the proportion of Veterans prescribed this medication with the projected phenotype or genetic variant.

[b]From eTable 8.

[c]Warfarin only has a strong recommendation for patients identifying as non-African ancestry. There are additional recommendations for patients with African ancestry, but we limited our analyses to the projected European ancestry proportion of our population. Therefore, 85% of the 205,177 unique warfarin users are projected to be of European ancestry in our model (n=174,400). Using the values that 4.0% of patients of European ancestry are projected to be *CYP2C9* poor metabolizers (eTable 4) and 65.2% of patients of European ancestry are projected to have actionable *VKORC1* actionable genotypes (eTable 3), we projected the % actionable phenotypes of patients of European ancestry using the following equation: *CYP2C9 poor*VKORC1 sens+CYP2C9 poor*VKORC1wt+VKROC1Sens*CYP2C9wt* = 0.04*0.652 + 0.04*(1-0.652) + (1-0.04)*0.652 = 66.6% of patients with European ancestry are projected to have actionable phenotype

© 2019 Chanfreau-Coffinier C et al. *JAMA Network Open.*

eFigure. Result of the Sensitivity Analysis for the Projected Prevalence of Actionable Genotypes Under Different Population Models

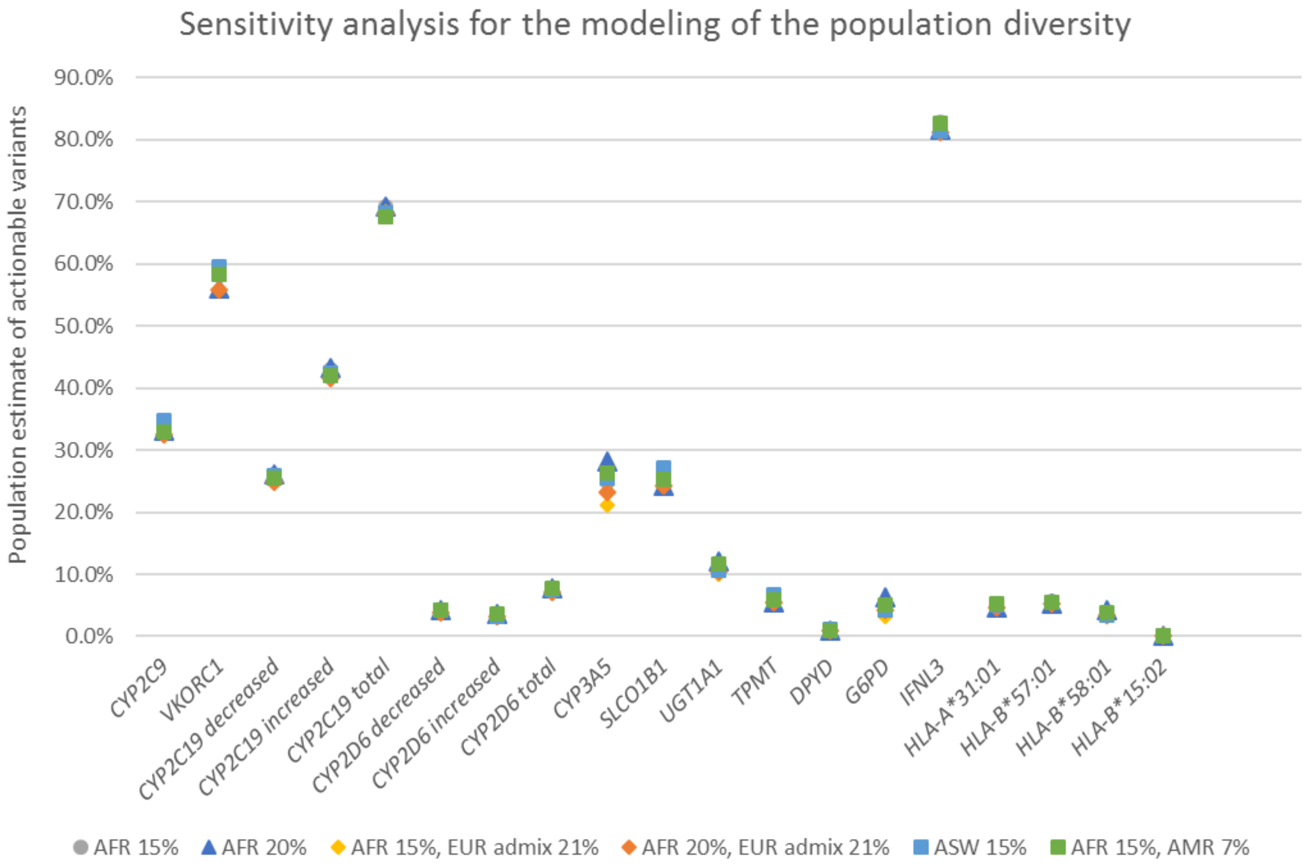

Abbreviations: AFR: African ancestry; AMR: Americas; ASW, People with African Ancestry in Southwest USA; EUR, European ancestry

Plot showing the distribution of estimates for the prevalence of PGX variants obtained under the six models (eTable 5)

AFR 15%, Model 1 with 15% patients of African ancestry and 85% of patients of European ancestry; AFR20%, Model 2, 20% patients of African ancestry and 80% of patients of European ancestry; AFR Models 3 and 4 models the admixture of African American patients with a 21% contribution of European ancestry; the 21% contribution was estimated based on the literature review summarized in eTable4. Model 5 applies the variant frequencies for the African American in Southwest USA in the 1000 Genome Project with weights of 15% ASW/85% EUR. Model 6 tests the effect of accounting the contribution of populations from American origin, and models the population as 15% for AFR, 7% AMR (population from Americas), and 78% EUR.

© 2019 Chanfreau-Coffinier C et al. *JAMA Network Open.*

eReferences.

1. Beoris M, Amos Wilson J, Garces JA, Lukowiak AA. CYP2D6 copy number distribution in the US population. *Pharmacogenet Genomics.* Feb 2016;26(2):96-99.
2. National Center for Veterans Analysis and Statistics. VA Benefits and Health Care Utilization. Washington, D.C, Department of Veterans Affairs, 2017. https://www.va.gov/vetdata/docs/pocketcards/fy2018q4.PDF.
3. *National Center for Veterans Analysis and Statistics. Minority Veterans Report: Military Service History and VA Benefit Utilization Statistics. Washington, D.C.: Data Goverance and Analytics, Department of Veterans Affairs, 2017.*
4. Delaney JT, Ramirez AH, Bowton E, et al. Predicting clopidogrel response using DNA samples linked to an electronic health record. *Clin Pharmacol Ther.* Feb 2012;91(2):257-263.
5. Caudle KE, Klein TE, Hoffman JM, et al. Incorporation of pharmacogenomics into routine clinical practice: the Clinical Pharmacogenetics Implementation Consortium (CPIC) guideline development process. *Curr Drug Metab.* Feb 2014;15(2):209-217.
6. Crews KR, Gaedigk A, Dunnenberger HM, et al. Clinical Pharmacogenetics Implementation Consortium guidelines for cytochrome P450 2D6 genotype and codeine therapy: 2014 update. *Clin Pharmacol Ther.* Apr 2014;95(4):376-382.
7. Birdwell KA, Decker B, Barbarino JM, et al. Clinical Pharmacogenetics Implementation Consortium (CPIC) Guidelines for CYP3A5 Genotype and Tacrolimus Dosing. *Clin Pharmacol Ther.* Jul 2015;98(1):19-24.
8. Phillips EJ, Sukasem C, Whirl-Carrillo M, et al. Clinical Pharmacogenetics Implementation Consortium Guideline for HLA Genotype and Use of Carbamazepine and Oxcarbazepine: 2017 Update. *Clin Pharmacol Ther.* Apr 2018;103(4):574-581.
9. Abecasis GR, Altshuler D, Auton A, et al. A map of human genome variation from population-scale sequencing. *Nature.* Oct 28 2010;467(7319):1061-1073.
10. Machiela MJ, Chanock SJ. LDlink: a web-based application for exploring population-specific haplotype structure and linking correlated alleles of possible functional variants. *Bioinformatics.* Nov 1 2015;31(21):3555-3557.
11. Johnson JA, Caudle KE, Gong L, et al. Clinical Pharmacogenetics Implementation Consortium (CPIC) Guideline for Pharmacogenetics-Guided Warfarin Dosing: 2017 Update. *Clin Pharmacol Ther.* Sep 2017;102(3):397-404.
12. Scott SA, Sangkuhl K, Stein CM, et al. Clinical Pharmacogenetics Implementation Consortium Guidelines for CYP2C19 Genotype and Clopidogrel Therapy: 2013 Update. *Clin Pharmacol Ther.* 2013;94(3):317-323.
13. Parra EJ, Marcini A, Akey J, et al. Estimating African American admixture proportions by use of population-specific alleles. *Am J Hum Genet.* Dec 1998;63(6):1839-1851.
14. Reiner AP, Ziv E, Lind DL, et al. Population structure, admixture, and aging-related phenotypes in African American adults: the Cardiovascular Health Study. *Am J Hum Genet.* Mar 2005;76(3):463-477.
15. Yaeger R, Avila-Bront A, Abdul K, et al. Comparing genetic ancestry and self-described race in african americans born in the United States and in Africa. *Cancer epidemiology, biomarkers & prevention : a publication of the American Association for Cancer Research, cosponsored by the American Society of Preventive Oncology.* Jun 2008;17(6):1329-1338.
16. Allison MA, Peralta CA, Wassel CL, et al. Genetic ancestry and lower extremity peripheral artery disease in the Multi-Ethnic Study of Atherosclerosis. *Vasc Med.* Oct 2010;15(5):351-359.
17. Murray T, Beaty TH, Mathias RA, et al. African and non-African admixture components in African Americans and an African Caribbean population. *Genet Epidemiol.* Sep 2010;34(6):561-568.
18. Bryc K, Durand EY, Macpherson JM, Reich D, Mountain JL. The genetic ancestry of African Americans, Latinos, and European Americans across the United States. *Am J Hum Genet.* Jan 8 2015;96(1):37-53.
19. Banda Y, Kvale MN, Hoffmann TJ, et al. Characterizing Race/Ethnicity and Genetic Ancestry for 100,000 Subjects in the Genetic Epidemiology Research on Adult Health and Aging (GERA) Cohort. *Genetics.* Aug 2015;200(4):1285-1295.
20. Baharian S, Barakatt M, Gignoux CR, et al. The Great Migration and African-American Genomic Diversity. *PLoS Genet.* May 2016;12(5):e1006059.
21. Mathias RA, Taub MA, Gignoux CR, et al. A continuum of admixture in the Western Hemisphere revealed by the African Diaspora genome. *Nature communications.* Oct 11 2016;7:12522.

© 2019 Chanfreau-Coffinier C et al. *JAMA Network Open.*

22. Hershfield MS, Callaghan JT, Tassaneeyakul W, et al. Clinical Pharmacogenetics Implementation Consortium guidelines for human leukocyte antigen-B genotype and allopurinol dosing. *Clin Pharmacol Ther.* Feb 2013;93(2):153-158.

23. Ramsey LB, Johnson SG, Caudle KE, et al. The clinical pharmacogenetics implementation consortium guideline for SLCO1B1 and simvastatin-induced myopathy: 2014 update. *Clin Pharmacol Ther.* Oct 2014;96(4):423-428.

© 2019 Chanfreau-Coffinier C et al. *JAMA Network Open.*
